# Effects of Plant Growth Hormones on *Mucor indicus* Growth and Chitosan and Ethanol Production

**DOI:** 10.3390/ijms160716683

**Published:** 2015-07-22

**Authors:** Zahra Safaei, Keikhosro Karimi, Poorandokht Golkar, Akram Zamani

**Affiliations:** 1 Department of Chemical Engineering, Isfahan University of Technology, 84156-83111 Isfahan, Iran; E-Mails: zs.safaie@ce.iut.ac.ir (Z.S.); karimi@cc.iut.ac.ir (K.K.); 2 Institutes of Biotechnology and Bioengineering, Isfahan University of Technology, 84156-83111 Isfahan, Iran; E-Mail: golkar@cc.iut.ac.ir; 3 Swedish Centre for Resource Recovery, University of Borås, 50190 Borås, Sweden

**Keywords:** chitosan, ethanol, glucosamine, *Mucor indicus*, plant hormones

## Abstract

The objective of this study was to investigate the effects of indole-3-acetic acid (IAA) and kinetin (KIN) on *Mucor indicus* growth, cell wall composition, and ethanol production. A semi-synthetic medium, supplemented with 0–5 mg/L hormones, was used for the cultivations (at 32 °C for 48 h). By addition of 1 mg/L of each hormone, the biomass and ethanol yields were increased and decreased, respectively. At higher levels, however, an inverse trend was observed. The glucosamine fraction of the cell wall, as a representative for chitosan, followed similar but sharper changes, compared to the biomass. The highest level was 221% higher than that obtained without hormones. The sum of glucosamine and *N*-acetyl glucosamine (chitin and chitosan) was noticeably enhanced in the presence of the hormones. Increase of chitosan was accompanied by a decrease in the phosphate content, with the lowest phosphate (0.01 g/g cell wall) being obtained when the chitosan was at the maximum (0.45 g/g cell wall). In conclusion, IAA and KIN significantly enhanced the *M. indicus* growth and chitosan production, while at the same time decreasing the ethanol yield to some extent. This study shows that plant growth hormones have a high potential for the improvement of fungal chitosan production by *M. indicus*.

## 1. Introduction

Due to the irreversible negative impacts of petroleum-based products on the environment as well as the limited petroleum resources available today, finding renewable alternatives for these products is among the strategic goals of various countries [1]. Biofuels and biopolymers are examples of the renewable materials, which play crucial roles in the development of sustainable societies for the future.

Indeed, ethanol is among the most important of biofuels, in terms of production volume. Nowadays, this biofuel is mainly produced by *Saccharomyces cerevisiae* [2]. However, there are some other microorganisms such as *Mucor indicus*, which produce ethanol with comparable productivities and yields. *M. indicus*, which was discovered several hundred years ago, is a zygomycete fungus that recently has received a lot of attention for ethanol production from lignocellulosic hydrolyzates. Simultaneous production of ethanol and chitosan from hexoses and pentoses is among the most important benefits of the fungus over *S. cerevisiae* [3,4,5].

Chitosan is a natural biopolymer with several applications in biotechnology, food, cosmetics, medicines, and waste-water treatment [6]. Despite the long-term industrial production of chitosan from shellfish wastes, production of this biopolymer from fungal resources, e.g., biomass of *M. indicus*, is still in the preliminary stages toward commercialization. During the exponential growth phase of zygomycetes, chitosan is produced and stored in the cell wall. Recovery and purification of chitosan is usually performed through subsequent alkali and acid treatments. Unlike the other fungal species, glucan is not available in the zygomycetes cell wall. In this group of fungi, chitosan, chitin, and polyphosphates are the main ingredients of the fungal cell wall while glucan is only available in fungal spores [3,6]. The biological production of chitosan has several benefits over the current industrial production through deacetylation of shellfish chitin. This includes milder production conditions, not being limited to seasonal supply, and the possibility to produce chitosan under completely controlled fermentation conditions [3,6,7].

Until now, there have been several studies trying to enhance the ethanol [8,9,10,11,12] or chitosan production by *M. indicus* [7,13,14,15]. However, fewer reports are available investigating the simultaneous changes of chitosan and ethanol production by this fungus under different conditions [4,5].

Mohammadi *et al.* [16], Chatterjee *et al.* [17], and Tan *et al.* [18] reported that chitosan production is significantly affected at different fungal growing conditions. Cultivation time, morphology of the growing fungus, and concentration of the different nutrients significantly alter the chitosan production by this fungus. However, much more investigation is still needed to understand all the factors influencing the fungal chitosan production.

Glucosamine (GlcN) is the dominant monomer in chitosan whereas *N*-acetyl glucosamine (GlcNAc) is the dominant monomer in chitin. In zygomycetes fungi biosynthesis of chitin and chitosan are strongly related. Biosynthesis of chitin is catalyzed by chitin synthase. In cytoplasm, this enzyme is packed in very small organelles called chitosomes. Once fusion of chitosome with plasma membrane takes place, chitin synthase is activated and chitin biosynthesis is started [15]. Upon fusion, chitin synthase can be located on plasma membrane in two different forms of associated and dispersed. The action of associated chitin synthase is ended with the formation of chitin microfibrils. In contrast, the dispersed chitin synthase units create separate needle like chitin chains, which can not be crystallized to microfibrils. This fraction of chitin undergoes the deacetylation reaction, by chitin deacetylase, and chitosan is formed. Therefore, the total amount of chitin and chitosan in the cell wall maybe enhanced if more chitin synthase units reach the plasma membrane. The chitin fraction (GlcNAc polymer) maybe increased if more chitin synthase units are in associated forms. In contrast more dispersed chitin synthase subunits would increase the chitosan (GlcN polymer) production [3,6,15].

The most common way to study the effects of different factors on chitosan production is to extract the chitosan from the cell wall of the fungus, cultivated under different growing conditions. Chatterjee *et al.* [17] investigated the effects of four types of plant growth hormones, by cultivation of the fungus at different concentrations of the hormones and extraction of chitosan from the cell wall using acetic acid solution. However, since the extraction conditions significantly affect the chitosan yield [6,19], in order to have a better understanding of the effect of the growing conditions on chitosan production by the fungus, an analytical method instead of an extractive one must be employed [20]. Furthermore, since chitosan is obtained as the byproduct of the ethanol production process by the fungus, it is interesting to study what effects different cultivation conditions have on simultaneous chitosan and ethanol production.

The objective of the current work was to investigate the effect of two types of plant growth hormones, indole-3-acetic acid (IAA) and kinetin (KIN), on chitosan and ethanol production by *M. indicus* in a semi-synthetic medium usually used for ethanol production. Chitosan content of the cell wall was estimated by accurate analysis of glucosamine and *N*-acetyl glucosamine contents in the cell wall. Besides, the changes in the cell growth, other cell wall ingredients, and protein content of the obtained fungal biomass were also investigated.

## 2. Results

In this study, a semi-synthetic culture medium usually used for the production of ethanol by *M. indicus*, was supplemented with different concentrations of two types of plant hormones from the auxin and cytokinin groups [5]. The ability of the fungus to grow at different hormone loadings was examined. Furthermore, the percentage of the protein and the cell wall in the fungal biomass was determined and compared at different hormone loadings. Moreover, the effect of the hormones on the chitosan content and other cell wall components was studied. Finally, changes in the production of the major metabolites of *M. indicus*, *i.e.*, ethanol and glycerol, were investigated.

### 2.1. Effects of Hormones on Biomass Production, Protein and Alkali Insoluble Material (AIM) Yields

According to Table 1, the biomass yield was influenced in the presence of indole-3-acetic acid (IAA). Adding 0.5 mg/L IAA to the control medium (medium with no hormones) resulted in 12% enhancement in the biomass yield. The maximum biomass production yield (0.09 g/g sugar) was achieved at 1 mg/L of IAA. At a higher concentration of IAA, however, the biomass yield was reduced (Table 1). The average protein yield was 0.56 g/g of the biomass; moreover, according to the standard deviations, changing the IAA concentration did not significantly affect the protein content (Table 1).

**Table 1:** The biomass, ethanol, glycerol, protein, AIM (Alkali Insoluble Material), phosphate, and sum of glucosamine (GlcN) and *N*-acetyl glucosamine (GlcNAc) in the presence of indole-3-acetic acid (IAA).

IAA (mg/L)	Biomass (g/g Sugar)	Ethanol (g/g Sugar)	Glycerol (g/g Sugar)	Protein (g/g Biomass)	AIM (g/g Biomass)	Phosphate (g/g AIM)	GlcN + GlcNAc (g/g AIM)
0	0.078 ± 0.001	0.39 ± 0.04	0.07 ± 0.00	0.58 ± 0.01	0.18 ± 0.00	0.09 ± 0.00	0.27 ± 0.04
0.5	0.087 ± 0.001	0.37 ± 0.05	0.07 ± 0.00	0.56 ± 0.01	0.18 ± 0.01	0.10 ± 0.00	0.50 ± 0.04
1	0.090 ± 0.001	0.20 ± 0.02	0.04 ± 0.00	0.53 ± 0.04	0.19 ± 0.00	0.01 ± 0.00	0.68 ± 0.04
2	0.087 ± 0.002	0.25 ± 0.01	0.04 ± 0.00	0.57 ± 0.01	0.16 ± 0.03	0.01 ± 0.00	0.56 ± 0.04
3	0.070 ± 0.002	0.28 ± 0.03	0.04 ± 0.00	0.56 ± 0.01	0.14 ± 0.02	0.07 ± 0.01	0.49 ± 0.02
5	0.069 ± 0.001	0.29 ± 0.00	0.07 ± 0.00	0.55 ± 0.01	0.16 ± 0.01	0.07 ± 0.00	0.42 ± 0.03

The AIM (Alkali Insoluble Material), the skeleton of the fungal cell wall, comprised 0.18 g/g biomass in the control treatment. Supplementation of the medium with 0.5–2 mg/L IAA did not change the AIM yield considerably. In contrast, the AIM yield was decreased at higher concentrations of this hormone (Table 1). The biomass yield exhibited similar changes in the presence of kinetin (KIN) (Table 2). Addition of 0.5 mg/L KIN was accompanied by a 9% enhancement in the biomass yield. This continued to increase at 1 mg/L KIN, where the maximum yield was obtained (0.09 g/g sugar). However, at higher concentrations, a decreasing style in the biomass yield was observed (Table 2). Nonetheless, there was no statistical difference in the protein yields at different KIN levels (Table 2).

The AIM yield was reduced in the presence of 0.5 mg KIN/l (from 0.18 to 0.15 g/g). However, the yield obtained at higher concentrations of this hormone was not statistically different from that obtained in the reference culture (Table 2).

**Table 2:** The biomass, ethanol, glycerol, protein, AIM, phosphate, and sum of GlcN and GlcNAc in the presence of kinetin (KIN).

KIN (mg/L)	Biomass (g/g Sugar)	Ethanol (g/g Sugar)	Glycerol (g/g Sugar)	Protein (g/g Biomass)	AIM (g/g Biomass)	Phosphate (g/g AIM)	GlcN + GlcNAc (g/g AIM)
0	0.077 ± 0.001	0.39 ± 0.04	0.07 ± 0.00	0.58 ± 0.01	0.19 ± 0.00	0.09 ± 0.00	0.27 ± 0.04
0.5	0.084 ± 0.001	0.32 ± 0.04	0.06 ± 0.00	0.56 ± 0.04	0.14 ± 0.01	0.01 ± 0.00	0.62 ± 0.03
1	0.090 ± 0.001	0.25 ± 0.00	0.04 ± 0.00	0.59 ± 0.04	0.19 ± 0.00	0.01 ± 0.00	0.54 ± 0.00
2	0.083 ± 0.002	0.34 ± 0.04	0.06 ± 0.00	0.56 ± 0.04	0.18 ± 0.00	0.09 ± 0.00	0.59 ± 0.02
4	0.076 ± 0.002	0.37 ± 0.02	0.07 ± 0.00	0.56 ± 0.02	0.17 ± 0.00	0.12 ± 0.00	0.45 ± 0.00
5	0.068 ± 0.002	0.42 ± 0.05	0.08 ± 0.00	0.55 ± 0.03	0.17 ± 0.00	0.11 ± 0.00	0.38 ± 0.00

### 2.2. Effects of Hormones on GlcN, GlcNAc and Phosphate

**Figure 1:**
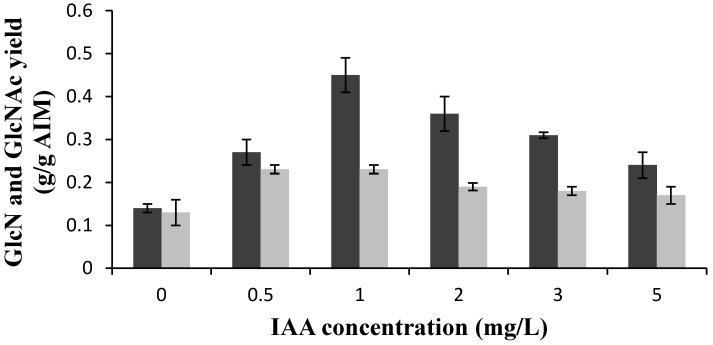
Effects of different concentrations of indole-3-acetic acid (IAA) (mg/L) on glucosamine (GlcN) (black bars) and *N*-acetyl glucosamine (GlcNAc) (gray bars) yields. Error bars represent the standard deviations of values obtained from at least two independent measurements.

In the absence of the hormones, glucosamine (GlcN) and *N*-acetyl glucosamine (GlcNAc) yields were 0.14 and 0.13 g/g AIM, respectively (Figure 1). The addition of 0.5 mg/L IAA resulted in a significant increase of the GlcN and GlcNAc yields (93% and 77%, respectively). Increasing the IAA concentration to 1 mg/L was accompanied by a considerable enhancement in the GlcN yield while GlcNAc was not affected significantly. The GlcN yield gradually decreased at higher concentrations of IAA, whereas the GlcNAc yield was almost constant. The highest GlcN yield was 0.45 g/g AIM, which was obtained at 1 mg/L IAA. At this hormone loading, the GlcN yield was three times higher than the yield obtained in the control medium (Figure 1). Therefore, the chitosan production was maximized at 1 mg/L of IAA, as its major building block, *i.e.*, glucosamine was its maximum at this concentration.

The sum of GlcN and GlcNAc in the AIM is an indication of total chitin and chitosan contents of the cell wall. According to Table 1, only 27% of the cell wall was composed of chitin and chitosan for the fungus grown in the control medium. The presence of IAA significantly enhanced the total chitin and chitosan content of the cell wall. At the highest achieved level (at 1 mg IAA/L), 68% of the cell wall of the *M. indicus* was comprised of GlcN and GlcNAc.

Besides GlcN and GlcNAc, phosphate was the other major fraction analyzed in the cell wall of the fungus. Phosphates comprised 9% of the AIM obtained in the control medium (Table 1). Addition of 0.5 mg/L of IAA did not have a significant impact on the phosphate yield. However, there was a sharp reduction in the phosphate yield by increasing the IAA concentration to 1 mg/L, at which the GlcN yield was maximized. The GlcN yield was decreased at 3 mg/L IAA and 5 mg/L IAA, while the phosphate yield was increased (Table 1). Generally, an inverse trend was observed for the GlcN and phosphate contents of the *M. indicus* cell wall.

**Figure 2:**
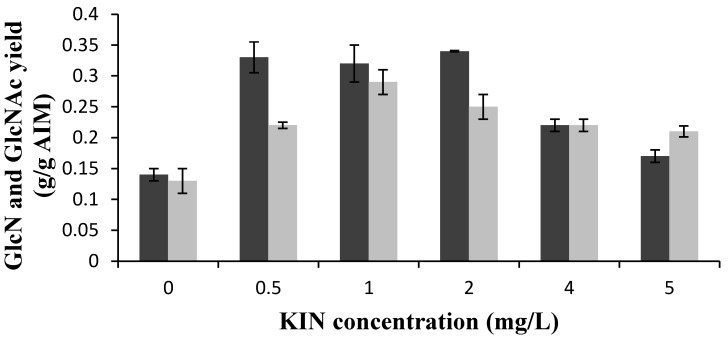
Effects of different concentrations of kinetin (KIN) (mg/L) on GlcN (black bars) and GlcNAc (gray bars) yields. Error bars represent the standard deviations of values obtained from at least two independent measurements.

Similar to IAA, the presence of KIN enhanced the GlcN yield. The yield increased more than twice (from 0.14–0.33 g/g AIM) in the presence of 0.5 mg/L of this hormone. However, statistically significant variations in the GlcN yield were not observed at 0.5–2 mg/L KIN. Higher concentrations of this hormone, however, reduced the GlcN yield. Similarly, using 0.5 mg/L KIN, improved the GlcNAc content by 123%. Further enhancement was achieved at 1 mg/L KIN where the GlcNAc yield was 0.22 g/g AIM. The GlcNAc yield, however, was decreased at higher concentrations of the hormone (Figure 2). Moreover, KIN significantly enhanced the total chitin and chitosan content of the fungal cell wall. The sum of the GlcN and GlcNAc content reached a maximum at 0.5 g/L KIN (0.62 g/g).

The phosphate content of the AIM was also significantly affected by adding KIN to the medium. Specifically, the phosphate content was decreased from 9% in the absence of the hormone to around 1% at 1–2 mg KIN/L where the GlcN content was at its maximum. In contrast, the phosphate content was enhanced to 11%–12% at 4–5 mg/L KIN where the GlcN yield was reduced (Table 2).

### 2.3. Effects of Hormones on Ethanol and Glycerol Yields

Ethanol and glycerol were the dominant metabolites in all the cultivations in the presence or absence of the hormones. In the control culture, after 48 h cultivation, the fungus produced 0.39 and 0.07 g ethanol and glycerol per g consumed sugar, respectively. The ethanol and glycerol yields were not significantly affected in the presence of 0.5 mg/L of IAA. However, 49% reduction in the ethanol yield was observed at 1 mg IAA/L where the lowest ethanol yield (0.2 g/g sugar) was obtained. At a higher concentration of IAA, however, increasing the hormone concentration gradually enhanced the ethanol yield. Comparable to ethanol, the glycerol yield was reduced by 43% by increasing the hormone concentration from 0.5–1 mg/L. The glycerol yield was also constant by increasing the hormone concentration from 1–3 mg/L. However, 75% enhancement in the glycerol yield was observed at 5 mg/L of the IAA (Table 1).

Generally, the ethanol and glycerol yields followed analogous trends in the presence of KIN and IAA. The ethanol yield was reduced from 0.39 g/g in the control culture to 0.32 g/g in the medium supplemented with 0.5 mg KIN/L. The yield continued to decline by increasing the hormone concentration and was minimum (0.25 g ethanol/g sugar) at 1 mg KIN/L. At higher concentrations, increasing the hormone concentration increased the ethanol yield. Similarly, the glycerol yield reached a minimum (0.04 g/g sugar) at 1 mg KIN/L (Table 2).

## 3. Discussion

Recently, the production of chitosan by zygomycetes fungi has been considered a promising alternative to the traditional chitosan production through the chemical deacetylation of crustaceans’ chitin. The possibility of enhancing the chitosan production by changing the cultivation conditions is among the most important positive features of this method. Among the different zygomycetes, the cell wall of *Mucor indicus* contains appreciable amounts of chitin and chitosan [3]. The results of the current study showed that plant hormones can significantly improve the fungal chitosan production. Moreover, the hormones considerably influence *M. indicus* growth, metabolite formation, and composition of the cell wall.

Mohammadi *et al.* [20] reported that the chitin and chitosan content of the cell wall in a filamentous form of this fungus was higher than yeast like morphology. Therefore, in this study, the fungus was grown with a filamentous form to achieve higher chitosan yields.

Not only the several steps of the plant growth, such as cell elongation, tissue swelling, cell division, and formation of adventitious roots are affected by the plant growth hormones [21], but also the growth of microorganisms and their cell composition can also be influenced by these hormones. Increase in the fresh weight and dry matter content of *Claviceps purpurea* mycelium, in the presence of indole-3-butyric acid and 2,4-dichloro phenoxy acetic acid, was reported by Rerabek [22]. Moreover, enhancement of biomass production and protein content of *Agaricus campestris* was achieved by adding 0.4 mg/L of IAA to the culture medium of the fungus [23]. Promoting the growth of a lichen-forming fungus, *Nephromopsis ornate*, 16.8%, in a malt-yeast extract liquid medium by adding 1 μM/L of IAA was reported by Wang *et al.* [24]. Additionally, fatal effects of IAA on *Nephromopsis ornate* at high concentrations, e.g., 10 and 50 μM/L of IAA, were also observed [24]. Similarly, the positive effect of the plant growth hormone (at concentrations up to 1 mg/L) on fungal growth as well as the negative effect of the hormones (at concentration higher than 1 mg/L) were observed in this study.

The enhancement of both mycelia growth and chitosan content of *Rhizopus oryzae*, in a whey medium at the optimum concentration of 3 and 2 mg/L IAA and KIN respectively, was reported by Chatterjee *et al.* [25]. Similar positive effects of these hormones on enhancement of growth of *M. indicus* and production of chitosan were observed in the current study. Furthermore, a toxic effect at a very high concentration of IAA and KIN resulted in a decrease in the biomass and chitosan production [25]. Although the plant growth hormones may enhance the protein content of fungal biomass [23], the results of this study showed no significant change in the protein content.

Addition of the plant growth hormones to molasses-salt culture of *M. indicus*, was also investigated by Chatterjee *et al.* [17]. They reported that 1 mg/L of IAA increased the mycelia growth and chitosan production by 14.7% and 36.4%, respectively. In this study, the biomass and chitosan yields were improved by 15% and 221% in the presence of a similar concentration of the hormone. In addition, the optimum concentration of KIN was 10 mg/L, according to Chatterjee *et al.*, at which the chitosan production increased by 42% [17]. In the current work, 128% increment in the chitosan yield was obtained at 1 mg/L of KIN (compared to the control medium). Therefore, a significantly higher improvement in the chitosan yield was obtained in the present study, compared to the work done by Chatterjee *et al.* [25]. On the other hand, higher concentrations of the hormones (higher than 1 mg/L) generally resulted in lower biomass and chitosan yields. Therefore, 1 mg/L of both KIN and IAA were chosen as the best concentration to enhance the chitosan production by the fungus. Moreover, further investigations are required to understand the effect of other plant hormones on the fungus growth and chitosan production.

In the current study, generally higher total chitin and chitosan contents were obtained in the presence of both of the hormones compared to the control medium (Table 1 and Table 2). This may indicate that the hormones facilitated the fusion of chitosomes to plasma membrane [3,15]. A significant improvement in GlcN yield was observed by increasing the IAA concentration from 0.5–1 mg/L while GlcNAc was almost constant (Figure 1). This might indicate that at this concentration the hormone facilitated the fusion of chitosomes mostly in dispersed form [3,15]. The same change in concentration of KIN (from 0.5–1 mg/L) showed a different effect and resulted in increasing the GlcNAc yield rather than the GlcN yield (Figure 2). Therefore, although the two hormones (0–5 mg/L) generally created comparable changes in GlcN and GlcNAc contents, they may have different mechanisms. Therefore, further investigations are required to understand the mechanism of the action of the hormones on fungal growth and cell wall development.

Generally, it is believed that chitosan and phosphates are directly connected to each other in the cell wall; therefore, higher phosphate yields are expected at higher chitosan contents [6,26]. However, the current results indicated that increasing the chitosan content of the cell wall of the fungus was accompanied by a reduction of the phosphate content. Therefore, the AIM yield was not affected significantly in the presence of the hormones while chitosan was increased and phosphate was decreased. However, further investigations such as studying the kinetics of cell growth and formation of cell wall in the presence of the plant hormones, are needed in order to understand the mechanisms of action of the hormones on the cell wall biosynthesis.

Ethanol production was affected in the presence of the hormones. Generally, lower ethanol yields were achieved when higher biomass and chitosan yields were obtained. The reverse trend of ethanol and biomass yields in ethanolic fermentations has been widely reported in the literature [3].

## 4. Experimental Section

### 4.1. Microorganism

The fungus *M. indicus* CCUG 22424 was obtained from the Culture Collection at the University of Gothenburg (Gothenburg, Sweden). The fungus was cultivated on agar plates containing (g/L) glucose 40, agar 20, and peptone 10, at 32 °C for 5 days. The obtained plates were used directly for the inoculation of the liquid media.

### 4.2. Plant Growth Hormones

Indole-3-acetic acid (IAA) and kinetin (KIN) (Sigma chemical Co., St. Louis, Mo, USA) were used in this study. The hormones were dissolved in sodium hydroxide solution (1 M). Hormone solutions were passed through a sterile filter (Millipore, Bedford, MA, USA, 0.22 μm) before addition to the culture.

### 4.3. Cultivation of the Fungus

An amount of 250 mL liquid medium containing (g/L): glucose, 50; KH_2_PO_4_, 3.5; (NH_4_)_2_SO_4_, 7.5; MgSO_4_·7H_2_O, 0.75; CaCl_2_·2H_2_O, 1; and yeast extract, 5 was prepared in 500 mL cotton plugged Erlenmeyer flasks. The pH was adjusted to 5.5, and the solution was autoclaved for 20 min at 121 °C. After cooling to room temperature, different concentrations (0–5 mg/L) of the sterile hormones (IAA or KIN) were added to the solution. Then, 10 mL of sterile distillated water was added to each agar plate to prepare the spore suspension. The concentration of this suspension was determined by direct counting using a Thoma lamella cell. Finally, all liquid cultures were inoculated with 1 mL of a spore suspension containing 2 × 10^5^ fungal spore/mL and incubated at 32 °C and 120 rpm for 48 h. At this spore concentration, fungal cells were grown in a purely filamentous form [3]. After 48 h, the obtained fungal biomass was separated by centrifugation (4000× *g* for 10 min), washed three times with distillated water, freeze dried, and stored at room temperature for further analysis.

### 4.4. Preparation of the Alkali Insoluble Material (AIM) and Determination of Glucosamine and N-Acetyl Glucosamine in the Cell Wall

The freeze-dried biomass was mixed with 0.5 M NaOH solution (30 mL/g biomass), and the mixture was treated at 120 °C for 20 min in an autoclave to prepare the alkali insoluble material (AIM) of the biomass. The AIM was then separated by centrifugation at 4000× *g* for 10 min and washed several times with distilled water until a neutral pH was reached. Finally, the AIM was freeze-dried and stored at room temperature until use. The AIM was analyzed as representative of the fungal cell wall [27]. Analysis of glucosamine and *N*-acetyl glucosamine was performed according to a method presented by Mohammadi *et al*. [20]. Briefly, 0.3 mL 72% (*v*/*v*) sulfuric acid was added to 10 mg AIM in 15 mL screw cap tubes. The suspensions were manually mixed for 90 min to dissolve the AIM in the concentrated acid. Then, 8.4 mL of water was added and the tubes were placed in an autoclave at 120 °C for 1 h to complete the sulfuric acid hydrolysis and convert the chitosan and chitin to glucosamine oligomers and acetic acid. After autoclaving, a sample (0.5 mL) was taken when the solution temperature was around 100 °C. This was allowed to cool down to room temperature, mixed with 0.5 mL of 1 M NaNO_2_, and left for 6 h at room temperature in a tightly closed tube. At the end of this step, the glucosamine oligomers were converted into 2,5-anhydromannose. Thereafter, the tubes were opened and placed under a hood overnight. To neutralize the unreacted nitrous acid, 0.5 mL of ammonium sulfamate (12% *w*/*v*) was added. Finally, the concentration of acetic acid and 2,5-anhydromannose, in sulfuric and nitrous acid hydrolyzed samples, respectively, were analyzed by high performance liquid chromatography (HPLC). The results were used for the calculation of GlcN and GlcNAc in the AIM, according to Mohammadi *et al*. [20].

### 4.5. Analysis of Sugar and Metabolites

After 48 h cultivation, the concentrations of the glucose, ethanol, and glycerol were measured in the media by High Performance Liquid Chromatography (HPLC, Jasco International Co., Tokyo, Japan). All HPLC analyses were conducted using an Aminex HPX87H column (Bio-Red, Hercules, CA, USA) with a refractive index detector (RI). Sulfuric acid solution (5 mM) with a flow rate of 0.6 mL/min at 60 °C was used as an eluent.

### 4.6. Determination of Proteins

The biuret method was used to determine the protein content [28]. The freeze-dried fungal biomass (0.1 g) was mixed with 3 mL 1 M NaOH solution for 2 h at room temperature. Then, the mixture was heated in boiling water for 10 min. Afterwards the sample was cooled down immediately in an ice bath and subsequently, 1 mL of 2.5% CuSO_4_·5H_2_O was added and mixed for 5 min. The mixture was finally centrifuged (3400× *g* for 4 min), and the absorbance of the supernatant was recorded at 555 nm.

### 4.7. Determination of Phosphates

The ammonium molybdate spectrometric method was used to measure the phosphate content according to European standard ISO6878 [29]. The supernatant obtained from the two-step sulfuric acid hydrolysis of the AIM, in the GlcN-GlcNAc determination test, (0.5 mL), was mixed with 70 mL distillated water. Then, 4 mL acid molybdate reagent and 4 mL ascorbic acid solution were added. Finally, the solution was diluted to 100 mL and the absorbance of the obtained blue color complex was measured at 880 nm (Shimadzu spectrophotometer, Model 240, Kyoto, Japan).

## 5. Conclusions

Chitosan production by *M. indicus* was considerably improved in the presence of 1 mg/L of the plant hormones KIN and IAA. Furthermore, increasing the chitosan yield reduced the phosphate yield in the fungal cell wall. Although the ethanol production was in reverse trend compared to the chitosan yield, ethanol was still the major metabolite formed during the cultivation of the fungus.

## References

[1] Ferreira J.A., Lennartsson P.R., Edebo L., Taherzadeh M.J. (2013). Zygomycetes-based biorefinery: Present status and future prospects. Bioresour. Technol..

[2] Taherzadeh M., Karimi K. (2008). Bioethanol: Market and production processes. Biofuels Refining and Performance.

[3] Karimi K., Zamani A. (2013). Mucor indicus: Biology and industrial application perspectives: A review. Biotechnol. Adv..

[4] Asachi R., Karimi K., Taherzadeh M. (2011). Fungal autolysate as a nutrient supplement for ethanol and chitosan production by *Mucor indicus*. Biotechnol. Lett..

[5] Heidary Vinche M., Asachi R., Zamani A., Karimi K. (2013). Ethanol and chitosan production from wheat hydrolysate by *Mucor hiemalis*. J. Chem. Technol. Biotechnol..

[6] Zamani A. (2010). Superabsorbent Polymers from the Cell Wall of Zygomycetes Fungi. Ph.D. Thesis.

[7] Davis L.L., Bartnickigarcia S. (1984). Chitosan synthesis by the tandem action of chitin synthase and chitin deacetylase from *Mucor rouxii*. Biochemistry.

[8] Abedinifar S., Karimi K., Khanahmadi M., Taherzadeh M.J. (2009). Ethanol production by *Mucor indicus* and *Rhizopus oryzae* from rice straw by separate hydrolysis and fermentation. Biomass Bioenerg..

[9] Karimi K., Brandberg T., Edebo L., Taherzadeh M. (2005). Fed-batch cultivation of *Mucor indicus* in dilute-acid lignocellulosic hydrolyzate for ethanol production. Biotechnol. Lett..

[10] Karimi K., Emtiazi G., Taherzadeh M.J. (2006). Ethanol production from dilute-acid pretreated rice straw by simultaneous saccharification and fermentation with *Mucor indicus*, *Rhizopus oryzae*, and *Saccharomyces cerevisiae*. Enzym. Microb. Technol..

[11] Sues A. (2003). Optimization of Ethanol and Biomass Production from Wood Hydrolysates by *Mucore indicus*. Master’s Thesis.

[12] Sharifia M., Karimi K., Taherzadeh M.J. (2008). Production of ethanol by filamentous and yeast-like forms of *Mucor indicus* from fructose, glucose, sucrose, and molasses. J. Ind. Microbiol. Biotechnol..

[13] Arcidiacono S., Kaplan D.L. (1992). Molecular weight distribution of chitosan isolated from *Mucor rouxii* under different culture and processing conditions. Biotechnol. Bioeng..

[14] Chatterjee S., Adhya M., Guha A.K., Chatterjee B.P. (2005). Chitosan from *Mucor rouxii*: Production and physico-chemical characterization. Process Biochem..

[15] Davis L.L., Bartnicki-Garcia S. (1984). The coordination of chitosan and chitin synthesis in *Mucor rouxii*. J. Gen. Microbiol..

[16] Mohammadi M., Zamani A., Karimi K. (2013). Effect of phosphate on glucosamine production by ethanolic fungus *Mucor indicus*. Appl. Biochem. Biotechnol..

[17] Chatterjee S., Chatterjee B.P., Guha A.K. (2007). Influence of plant growth hormones on the growth of *Mucor rouxii* and chitosan production. Microbiol. Res..

[18] Tan S.C., Tan T.K., Wong S.M., Khor E. (1996). The chitosan yield of zygomycetes at their optimum harvesting time. Carbohydr. Polym..

[19] Naghdi M., Zamani A., Karimi K. (2014). A sulfuric–lactic acid process for efficient purification of fungal chitosan with intact molecular weight. Int. J. Biol. Macromol..

[20] Mohammadi M., Zamani A., Karimi K. (2012). Determination of glucosamine in fungal cell walls by high-performance liquid chromatography (HPLC). J. Agric. Food Chem..

[21] Tingwa P., Young R. (1975). The effect of indole-3-acetic acid and other growth regulators on the ripening of avocado fruits. Plant Physiol..

[22] Rerabek J. (1970). Influence of auxins on growth of *Claviceps purpurea* (fries) tulasne in saprophytic cultures. Folia Microbiol..

[23] Guha A.K., Banerjee A.S. (1974). Effect of indole-3 acetic acid and kinetin on submerged growth of *Agaricus campestri*. Acta Microbiol. Pol. B.

[24] Wang X.Y., Wei X.L., Luo H., Kim J.A. (2010). Plant hormones promote growth in lichen-forming fungi. Mycobiology.

[25] Chatterjee S., Chatterjee B.P., Guha A.K. (2008). Enhancement of growth and chitosan production by *Rhizopus oryzae* in whey medium by plant growth hormones. Int. J. Biol. Macromol..

[26] Zamani A., Edebo L., Sjostrom B., Taherzadeh M.J. (2007). Extraction and precipitation of chitosan from cell wall of zygomycetes fungi by dilute sulfuric acid. Biomacromolecules.

[27] Zamani A., Jeihanipour A., Edebo L., Niklasson C., Taherzadeh M.J. (2008). Determination of glucosamine and *N*-acetyl glucosamine in fungal cell wall. J. Agric. Food Chem..

[28] Verduyn C., Ostma E., Chheffers W.A., Dijken J.P. (1990). Physiology of *Saccharomyces cerevisiae* in anaerobic glucose-limited chemostat culture. J. Gen. Microbiol..

[29] Zamani A., Edebo L., Niklasson C., Taherzadeh M.J. (2010). Temperature shifts for extraction and purification of zygomycetes chitosan with dilute sulfuric acid. Int. J. Mol. Sci..

